# Supplementary material for: Integrative Network Fusion: A Multi-Omics Approach in Molecular Profiling
Source: Front Oncol. 2020 Jun 30;10:1065. doi: 10.3389/fonc.2020.01065 (PMC7340129; doi:10.3389/fonc.2020.01065)

Supplementary Material

1 SUPPLEMENTARY TABLES AND FIGURES

1.1 Figures

Figure S1. UMAP projections on the BRCA-subtypes task with 3-layer juxtaposed data. Each subplot represents the projection of the TR/TS/TS2 partition for the remaining 9 splits not reported in the main text. Circle: TR set; triangle: TS set; diamond: TS2 set.

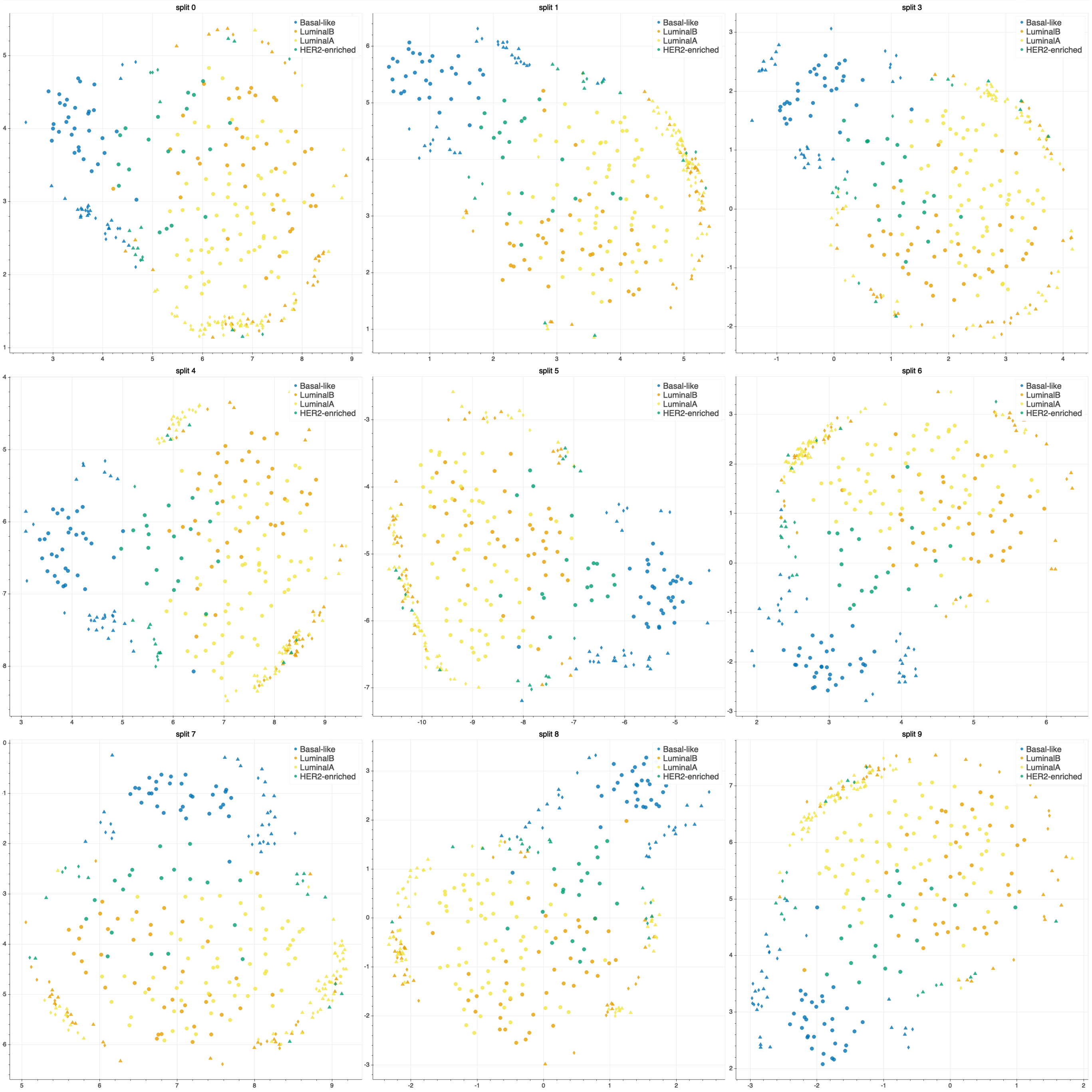

Figure S2. UMAP projections on the BRCA-subtypes task with 3-layer juxtaposed data restricted to the INF signature. Each subplot represents the projection of the TR/TS/TS2 partition for the remaining 9 splits not reported in the main text. Circle: TR set; triangle: TS set; diamond: TS2 set.

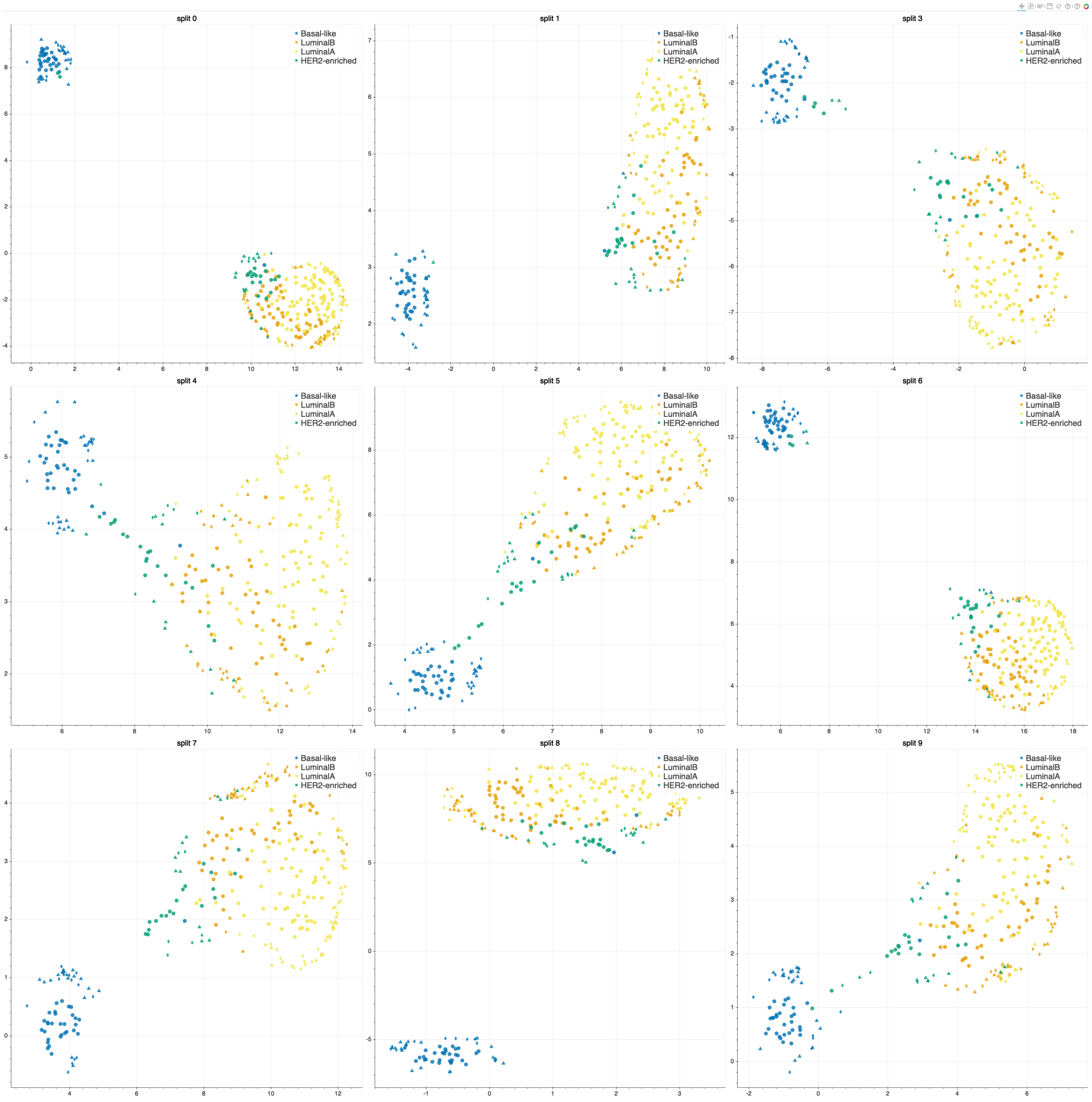

Supplement: Supplementary file 2 [file Data_Sheet_1.pdf]
